# Ceruloplasmin (2-D PAGE) Pattern and Copper Content in Serum and Brain of Alzheimer Disease Patients

**Published:** 2007-02-07

**Authors:** Rosanna Squitti, Carlo C. Quattrocchi, Gloria Dal Forno, Piero Antuono, David R. Wekstein, Concetta R. Capo, Carlo Salustri, Paolo M. Rossini

**Affiliations:** 1 AFaR, Dept. of Neuroscience, Fatebenefratelli Hospital, Rome, Italy; 2 Depts. of Radiology “Campus Biomedico” University, Rome, Italy; 3 Depts. of Neurology, “Campus Biomedico” University, Rome, Italy; 4 Dept. of Neurology, Medical College of Wisconsin, Milwaukee, WI, U.S.A; 5 University of Kentucky Alzheimer’s Disease Research Center, Lexington, KY, U.S.A; 6 Dept. of Biology, Tor Vergata University, Rome, Italy; 7 Institute of Cognition Sciences and Technologies (CNR), Rome, Italy; 8 IRCCS ‘Centro S. Giovanni di Dio-FBF’, Brescia, Italy

**Keywords:** Alzheimer’s disease, copper, ceruloplasmin, serum, brain, SDS-PAGE

## Abstract

A dysfunction in copper homeostasis seems to occur in Alzheimer’s disease (AD). We previously evidenced that an excess of non-ceruloplasmin-copper (NCC) correlated with the main functional, anatomical as well as cerebrospinal markers of the disease. Aim of our study was to investigate ceruloplasmin isoforms as potential actors in this AD copper dysfunction. Our data show that AD patients have ceruloplasmin fragments of low molecular weight (<50 kDa) both in their serum and brain, contrary to healthy controls. Ceruloplasmin isoforms of higher molecular weight (115 and 135 kDa in serum and 135 kDa in brain), as well as copper levels in the brain, instead, do not seem to mark a difference between AD and healthy subjects. These data suggest a ceruloplasmin fragmentation in the serum of AD patients. Some clues in this direction have been found also in the AD brain.

## Introduction

Alzheimer’s Disease (AD) is a heterogeneous, progressive neurodegenerative disorder representing the most common cause of dementia in the elderly. There is compelling evidence that in this disease beta amyloid (Aβ) deposition triggers oxidative stress as well as anomalous metal—Aβ protein interaction. Recent studies have shown that metals such as copper, iron and zinc are key mediating factors in these processes. High concentrations of copper and iron are found within senile plaques and neurofibrillary tangles of AD brains (Smith et al. 1997; Lovell et al. 1998; Sayre et al. 2000). Both metals can catalyze Fenton’s reactions, generating a flux of reactive oxygen species that can potentially damage functional and structural macromolecules (Smith et al. 1997). Moreover, the amyloid precursor protein (APP) is a crucial regulator of neuronal copper homeostasis (Barnham et al. 2003) and an abnormal brain homeostasis of metals could contribute to set up chemical conditions where toxicity and deposition of Aβ are promoted (Bush, 2002). These phenomena could be due simply to aging or to variations in levels of circulating blood copper (Bush, 2002). In fact, recent evidence showed that in a model of cholesterol fed rabbits, the ingestion of ≈2 μM of copper in drinking water markedly changed the brain β-amyloid burden with no variation of serum ceruloplasmin levels. This suggests that ingested copper supplied the serum exchangeable copper pool (Sparks and Schreurs, 2003).

The debate on the toxic or protective role of copper in AD is still ongoing. In opposition to some studies supporting copper toxic role (White et al. 1999; Huang et al. 1999) and a copper elevation in AD (Squitti et al. 2002; Ritchie et al. 2003; Smorgon et al. 2004; Squitti et al. 2004; Bomboi et al. 2005; Squitti et al. 2006), new studies support the notion of a protective role of this metal (Bayer et al. 2003; Phinney et al. 2003; Pajonk et al. 2005; Kessler et al. 2006). Moreover, differences in ceruloplasmin and cholesterol levels were observed between AD patients and controls supporting a role of cholesterol in AD and a possible influence of increasing circulating copper levels (Sparks et al. 2005). Actually, copper distribution in AD is very complex. Indeed, new evidence suggests that copper in brain may redistribute outside the cell, leaving the cell relatively deficient (Religa et al. 2006).

Copper is a cofactor of several intracellular enzymes, including cytochrome oxidase, copper-zinc superoxide dismutase and lysyl oxidase (Gubler et al. 1952). Over 90% of copper contained in human serum is considered bound to ceruloplasmin, making this protein a central constituent of copper transport and metabolism. Although past studies in man have found no differences in serum copper levels between AD and controls (Molina et al. 1998; Ozcankaya and Delibas, 2002), more recent studies have shown both an increase (Gonzàlez et al. 1999; Squitti et al. 2002; Ritchie et al. 2003; Smorgon et al. 2004; Bomboi et al. 2005, Squitti et al. 2006) and a decrease of these levels (Pajonk et al. 2005; Kessler et al. 2006). This seemingly bi-directional behavior is probably due to the fact that absolute levels of serum copper are considered: when only the non-ceruloplasmin-copper (NCC) fraction is considered, discrepancies are actually attenuated. For example, a value of 4 μM of NCC can be calculated from data reporting low levels of total serum copper in AD (Kessler et al. 2006). This is substantially higher than 1.6 μM, which represents the upper value of the NCC normal reference range in healthy populations (Hoogenraad, 2001). This suggests that it is the ceruloplasmin-copper relationship that represents the key to interpret copper findings in AD living patients, rather than absolute serum copper levels. It is upon this assumption that we focused our study on the alterations in the protein structure and on the composition of the whole ceruloplasmin protein as isoforms, expressing pattern changes or protein post-transductional modifications (Brenner, 2003; Squitti et al. 2005 and 2006).

We had already demonstrated that the loosely bound copper fraction (NCC) is specifically higher than normal in AD patients (Squitti et al. 2005), that it has blood brain barrier filtering properties, and that it correlates with markers of neurodegeneration of AD in the CSF, namely β-amyloid, h-tau and p-tau (Squitti et al. 2006). We report here on the qualitative changes of ceruloplasmin in the serum of AD patients explored by means of a two dimensional polyacrylamide gel electrophoresis (2-D PAGE) approach. We also report on a preliminary investigation on ceruloplasmin in AD brain tissues.

## Materials and Methods

The study was approved by the local IRB and all participants or legal guardians signed an informed consent. Brain tissues from AD patients were kindly provided by the Brain Bank of the Medical College of Wisconsin, USA and those from control cases by the Biologically Resilient Adults in Neurological Studies (BRAINS) project (Schmitt et al. 2001).

### Tissues

Serum and brain samples were obtained from different AD patients, as they could not be obtained from the same subject. *Serum*—samples of fresh serum were obtained from 25 individuals with a diagnosis of probable AD, (NINCDS-ADRDA criteria) (McKhann et al. 1984) and a Mini-Mental State Examination (MMSE) (Folstein et al. 1975) score of 25 or less (ages 65–81 years, mean = 75 years), and 25 age- and sex-matched cognitively normal individuals (ages 64–89 years, mean = 76.1 years), MMSE (mean = 28). Criterion for exclusion of both patients and controls was presence of conditions affecting copper metabolism and biological variables of oxidative stress (e.g. diabetes mellitus, inflammatory diseases, Hodgkin’s disease, recent history of heart or respiratory failure, chronic liver or renal failure, malignant tumors, alcohol abuse). Serum samples from AD patients and controls were pooled. Ceruloplasmin content in both pools was made to 28 mg/dL and each serum sample contributed equally to ceruloplasmin content. Serum pools were precipitated in an 8:1 v/v 100% methanol. The samples were kept at −20° C for 2 h and then centrifuged at 13000 rpm for 30 min at 4°C. The supernatant was discarded and the pellet resuspended in 450 μl of a solution for isoelectric focusing (IEF) (2D PAGE sample buffer), containing 8 M urea, 2% CHAPS, 0.3% DTT, 0.5% Ampholine pH 3.5–10 and traces of bromophenol blue.

Tissues from the cerebral cortex, including temporal neocortex, were obtained from 9 cases of AD NINCDS-ADRDA (McKhann et al. 1984) (ages 80–91 years, mean = 84.6; postmortem interval (PMI) 1–7.5 h, mean = 4.3 h), confirmed histopathologically at autopsy (Khachaturian, 1985), along with 10 control cases (ages 73–87 years, mean = 80.2 years; PMI 1–6 h, mean 3.6 h). Neuritic plaques were counted on silver stained serial sections according to previously reported methods (Yamamoto and Hirano, 1986).

### Preparation of brain samples for SDS PAGE or 2D-PAGE analyses

Brain samples from the associative cortex (area 9), were weighted, homogenized in a 3× w/v (10mM HEPES, 10mM NaCl, 1mM KH_2_PO_4_, 5mM NaHCO_3_, 1mM CaCl_2_, 0.5mM MgCl_2_; 4°C) containing a protease inhibitor mixture (2 mg/mL leupeptin, 1 μg/mL pepstatin, 0.1 mg/mL PMSF) and sonicated with 10 strokes at 4°C (IKA LABORTECHNIK). Protein concentration was determined using a Dc Protein Assay kit (Bio-Rad Laboratories, Hercules, CA, U.S.A.) based on Lowry et al. (1951). Brain homogenate aliquots with equal protein content from AD and control sample were pooled separately. Homogenates were, subsequently, fractionated using a previously reported method (Guillemin et al. 2005): briefly, 0.1 volumes of a 2.5M sucrose solution was added to the pool, centrifuged at 11000 rpm for 10 min at 4°C, the pellet stored at −80°C and the supernatant centrifuged again at 14000 rpm (3 h at 4°C). The second supernatant was desalted, then dehydrated using a refrigerated SpeedVac Plus centrifuge (ThermoSavant Instruments Inc., Holbrook, NY, U.S.A.), and finally resuspended in either SDS PAGE or 2D-PAGE sample buffer and stored at −80°C.

### Copper and ceruloplasmin content measurements

Serum copper concentration was measured by both methods of Abe et al. (1989) (Randox Laboratories, Crumlin, U.K.), and an A Analyst 300 Perkin Elmer atomic absorption spectrophotometer equipped with a graphite furnace with platform HGA 800. Serum ceruloplasmin was measured by immunoturbidimetric assay using a rabbit anti-human ceruloplasmin antibody in phosphate buffer (Roche, Diagnostic, Germany). Copper estimation by the Abe’s method and ceruloplasmin measures were automated on a Hitachi 917 analyser (Roche Diagnostics) and performed in triplicate. For each serum copper and ceruloplasmin pair contributing to the AD and control pool, the copper bound to ceruloplasmin (CB) and the loosely bound fraction, referred to as non-ceruloplasmin-bound copper (NCC), were calculated. Copper bound to ceruloplasmin was computed as follows (Walshe, 2003): CB = n * ceruloplasmin mg/L; n = 0.0472 (μmol/mg). By subtracting CB from the total serum copper, the “non ceruloplasmin bound copper” (NCC) value was estimated (Stremmel et al. 1991; Walshe, 2003; Twomey et al. 2005).

For brain copper quantitation, frozen brain specimens were weighted and homogenized in a 3× w/v. A hundred μl of brain extracts were diluted to 150 μl of dH_2_O and an equal volume (150 μl) of a 65% nitric acid solution was added. After 1 week of digestion, atomic absorption was measured by an atomic absorption spectrophotometer.

### Electrophoresis and Immunoblotting

SDS-PAGE were performed on 10% SDS-polyacrylamide separating gels, run at constant 15 mA (15′) through the stacking gel, and at constant 30 mA through the separating gel. Proteins were transferred to nitrocellulose membranes (Bio-Rad Laboratories, CA, U.S.A.) at 4°C (60 V, 2h), blocked in TBS containing 0.2% Tween-20 and 10% non-fat dry milk (1h at room temperature) and incubated with a 1:10,000 goat anti-human ceruloplasmin antibody solution (1h at RT) (Sigma-Aldrich Corporation, St. Louis, MI, U.S.A.). Films were developed by incubating membranes with 1:80,000 HRP-conjugated anti-goat secondary antibody solution and using an enhanced luminol reagent (Perkin Elmer Life Sciences, Boston, U.S.A.). Kodak films were used.

2-D PAGE were performed on a Protean IEF Cell (Bio-Rad Laboratories, CA, U.S.A.) for about 25 KVh after the dry 17 cm pH 4.5–5.7 and the 7cm pH 3–10 IPG strips (Bio-Rad Laboratories, CA, U.S.A.) were rehydrated with the sample solution; the strips were then equilibrated and run on a second dimension 10% or 7–20% gradient polyacrylamide gel. Proteins were transferred to nitrocellulose membranes and developed with a specific polyclonal anti-human ceruloplasmin as described above. Four gel replicates of each pooled sample per experiment were performed and developed on the same film to avoid procedural bias in the optical density reading (intra-run variation assay). Then, three sets of experiments were performed to assess the inter-run variation. Analysis of 2D PAGE films was performed by using PDQuest 2-D analysis software (Bio-Rad Laboratories, CA, U.S.A.), creating a master map from match data sets of 4 replicates and comparing optical density of AD pool vs. control pool. The same procedure analysis was followed on serum and brain samples.

## Results

### Copper concentration in the serum of patients with AD is higher than controls

In Table 1, levels of serum copper biological variables expressed as mean (SD) of individual concentrations contributing to patient or control pools, are reported. Serum copper in the AD group was higher (p < 0.001) while differences in ceruloplasmin and copper bound to ceruloplasmin concentrations between AD and control samples were not evident (p = 0.369). The amount of the calculated NCC was higher in AD (test t, p = 0.001) when compared to controls.

### Ceruloplasmin 2D map in serum of AD patients and controls

**Figure 1:**
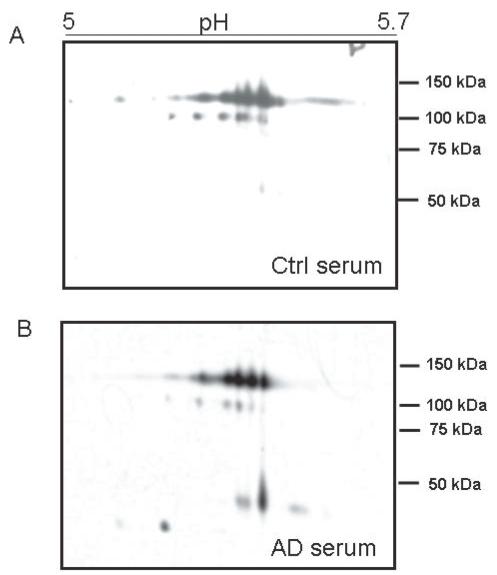
2D analysis of serum ceruloplasmin in AD (**A**) and control (**B**). The pH 5–5.7 range is shown. Note how similar is the protein pattern at about 135 and 115 kDa. Low molecular weight (<50 kDa) positive spots are present only in AD serum (**B**). 450 μl of a 1.5 mg/mL protein solution were loaded per gel. Ceruloplasmin content in both pools was made to 28 mg/dL and each serum sample contributed equally to the ceruloplasmin content.

**Figure 2:**
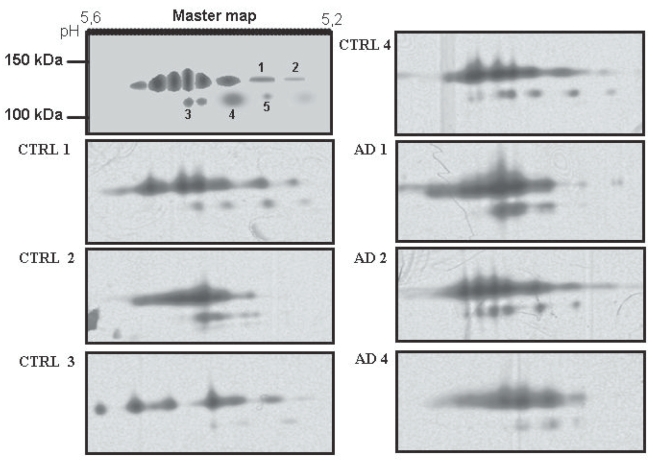
2D quantitative analysis of serum ceruloplasmin isoforms at 135 and 115 kDa. A master map created by using PDQuest software from a triplicate of AD sample and a quadruplicate of control sample. The optical density analysis of the spots did not show statistically significant differences between AD and control sample.

To reveal qualitative isoform differences in serum ceruloplasmin between AD and controls, 2-D PAGE experiments were carried out by pooling sera weighted for the same amount of ceruloplasmin (28 mg/dL) per sample. No gross changes were revealed for the 135 and 115 kDa molecular weight forms (Figure 1). The immunoblotting 2-D maps showed various spots in the pH 4.5–pH 5.7 range, resolving the 135 and the 115 kDa band (Figure 1), usually detected on ceruloplasmin SDS-PAGE. The main difference between AD and control ceruloplasmin was the presence of lower molecular weight spots (<50 kDa) in the AD, not detected in control serum. Analysis of the separated 135 kDa and 115 kDa spots, identified on a master map created by using the PDQuest analysis software, showed no qualitative differences for spots composing of the 135 and 115 kDa forms, suggesting that the spectrum of ceruloplasmin isoforms was substantially superimposable between AD and control samples (Figure 2). By 2-D PAGE analysis, it was possible to identify 8 spots at 135 kDa and 5 ones at 115 kDa. Quantitative analysis of optical density of the separated spots showed a relative >50% different density in five identified spots labelled 1 to 5 in Figure 2. The ceruloplasmin spots 1, 2 and 5 were decreased, while spots 3 and 4 were increased in AD of at least 50%. However, the variability experienced during different trials allowed us to rule out a significant relevance of these differences.

### Ceruloplasmin is detected in brain samples from AD and control subjects

**Figure 3:**
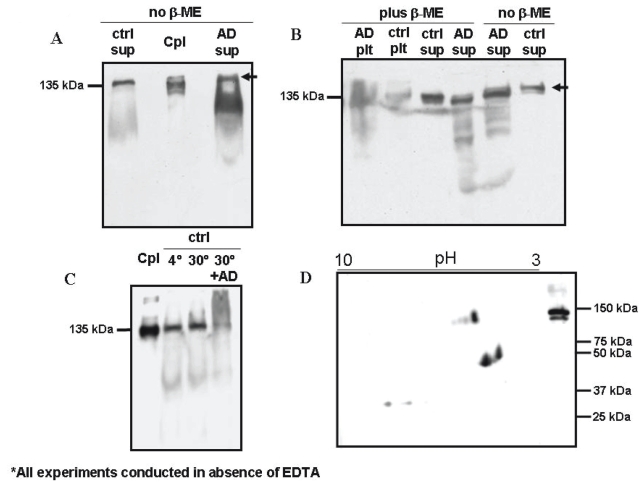
Brain ceruloplasmin shows proteolysis products in AD sample. **A.** Monodimensional SDS-PAGE performed in the absence of β-mercapto-ethanol shows a higher amount of the 115 kDa isoform in the AD sample. A ≅ 200kDa band is indicated by the arrow; **B.** Same as Panel A in the presence of β-mercapto-ethanol shows disappearance of the ≅ 200 kDa band, lower molecular weight fragments in the AD sample and presence of ceruloplasmin in cytosol and membrane fractions; **C.** Control sample shows a sharp band at the expected MW, even after 3 hours incubation at 30°C, whereas a smear at higher molecular weight appears when it is incubated in presence of 1/10 of the AD sample; **D.** 2D map of a brain extract from AD patients shows few spots at the full-length MW and some intense spots at lower molecular weight (<50 kDa). Cpl, ceruloplasmin; ctrl, control; AD, Alzheimer disease; plt, membrane fraction; sup, cytosol fraction.

The SDS PAGE study of brain extracts was developed in 2 steps. In a preliminary one, AD and control samples were run following standard SDS PAGE protocols. Using this procedure both samples showed a band at a high molecular weight, named 200 kDa band (Figure 3A). Subsequently, strong reducing conditions were used (2 μl of beta-mercaptoethanol incubation 5 minutes before loading), resulting in the disappearance of the 200 kDa, and the resolution of the 135 kDa band (Figure 3B). In the control sample the 135 kDa was the only band resolved, while in the AD sample, few lower molecular weight bands were evident (Figure 3B). Low molecular weight bands in the AD brain sample might be referred to AD proteolytic processes occurring in the living patient or to processes of post-mortem or sample storage degradation. To assess if low molecular weight bands in the AD brain specimen could be referable to processes of degradation specific of AD, the control sample was incubated 3 h at 30°C with 1/10 v/v of the AD sample. In this case, low molecular weight fragments of ceruloplasmin in the control pool were not generated. Instead, a high molecular weight smear was visualized even at strong reducing conditions, possibly accounted for the formation of high molecular weight agglomerates in the presence of AD ceruloplasmin fragments. The control sample alone in the same conditions did not show either degradation products or high molecular weight agglomerates (Figure 3C).

Pellet samples also showed a relative amount of membrane-bound ceruloplasmin (Fig. 3B).

Two-dimension PAGE experiments carried out on AD brain samples detected 4 spots at 135 kDa, and some smaller fragments in the 40–50 kDa range, reproducing the evidence of the 2D PAGE of serum ceruloplasmin (Musci et al. 1993). In the brain specimens of both AD and controls the 115 kDa isoforms, characteristic of the ceruloplasmin in serum, were not detected. The same experiments performed on control brains did not successfully show interpretable spots (Fig. 3D).

The average of the neuritic plaques load counted in AD specimens was 33 (5.6). Copper was also separately quantified in each cortical tissue sample, and did not differ between the two groups (Table 2).

## Discussion

The main result of our study was the identification of fragments of ceruloplasmin proteolysis both in the serum and in the brains of AD patients. The evidence of ceruloplasmin fragmentation suggests that at least a portion of ceruloplasmin is present as protein apoform and that impairment in the incorporation of copper into the protein may occur during its biosynthesis. This seems to be the main change in the ceruloplasmin structure: our data demonstrate that the ceruloplasmin isoform profile is not qualitatively different in AD patients than in healthy controls in terms of molecular weight and protein isoelectric pH. However, alternative hypotheses could be put forward to explain ceruloplasmin fragmentation. In particular, it could be caused by oxidative insult as a result of incorrect or overloading of copper into the protein, or an up regulation of redox active enzymes causing a general increase in oxidative stress or a down regulation in enzymes that normally regulate oxidative stress; both forms of enzyme would be susceptible to changes in copper homeostasis as has been reported to occur in Alzheimer’s disease. Further studies are needed to explore these alternative hypothesis even though the limited amount of AD patients samples is an important limiting factor.

Ceruloplasmin apoprotein is excessively secreted by hepatocytes in the presence of copper incorporation impairment, because of a dysfunction of the copper–transporting ATPase 7b, as it occurs in Wilson’s disease (Bielli and Calabrese, 2002). The idea of an impairment of copper incorporation into the ceruloplasmin protein in AD comes from the notion that the ceruloplasmin apoprotein is rapidly degraded in plasma (Bielli and Calabrese, 2002). The presence of ceruloplasmin fragments <50 kDa in AD suggests a similarly impaired copper transfer into the secretory pathway of hepatocytes. Ceruloplasmin degradation could, then, partially account for the rise of the NCC component. Even if the NCC mean value in AD reaches a level 9 times lower than that found in Wilson’s disease, it is still sensibly higher than the value found both in our controls and in the general healthy population (0–1.6 μmol/L) as also arguable from data presented by other authors (Kessler et al. 2006). Moreover, we recently obtained preliminary data of a filtered unbound copper component in AD 3.7 times higher than the value in healthy controls (Squitti et al. 2006). Both ultrafiltration and the 2D-PAGE data support an impairment of copper incorporation into ceruloplasmin and a possible defect in biliary copper excretion, as some authors have suggested (Bush, 2004).

The profile of ceruloplasmin isoforms at 115 and 135 kDa in serum reported in the present study is consistent with previous evidence in normal aging (Musci et al. 1993), and the isoforms of the 135 and 115 KDa bands were qualitatively similar in AD and controls.

The low MW bands observed in the brain suggest that ceruloplasmin fragmentation may occur also in the brain of AD patients. In our experiment, where a brain control sample was incubated for 3 h at 30°C together with 1/10 v/v of the AD sample, no ceruloplasmin proteolysis took place, suggesting that the fragmentation is not due to processes of post-mortem or sample storage degradation, but rather to AD-related degeneration processes, which took place during the patient’s life. Moreover, our experiment allowed to mark the moment in the ceruloplasmin lifetime when the fragmentation took place. In fact, the control sample did not show any fragmentation after the incubation, indicating that the AD-related degradation did not act upon a properly formed ceruloplasmin molecule but rather upon its apoform, i.e. during its biosynthesis.

The 115 kDa band was not detectable in the brain tissue, either with SDS PAGE or 2D-PAGE. Our findings are in line with results previously reported for the Glycosylphosphatidylinositol-anchored (GPI) form of ceruloplasmin, synthesized in mammalian astrocytes (Patel and David, 1997). Indeed these authors revealed an electrophoretic band at the MW of 135 kDa for the GPI form.

When we carried out the SDS PAGE resolution of ceruloplasmin from brain samples with standard procedures, we evidenced a band at 200 kDa MW, both in AD and in control specimens, which has been reported by at least one author (Sato et al. 1990). However, when these two fractions were further analysed by denaturing SDS-PAGE, the 200 kDa band was no more detectable, as also described by Sato and colleagues (1990), and only the 135 kDa band could be resolved in both samples (Figure 3B). These findings suggest that the band at high MW may be the result of ceruloplasmin chain aggregation of the apparent weight of 200 kDa.

2-D PAGE resolved the 135 kDa MW band in brain samples in 4 distinct isoforms of ceruloplasmin. Our data extend previous evidence of a single polypeptide species, identified as the GPI anchored form of ceruloplasmin (Patel and David 1997). In fact, we used a polyclonal antibody with a broad spectrum of antigen affinity, which might account for the larger number of isoforms we found.

The difficulties we experienced in detecting ceruloplasmin in 2D experiments with controls—in which we started from brain crude extracts—under the same conditions as in AD, suggest that the amount of ceruloplasmin in AD was higher than in control brains, in agreement with previous literature (Loeffler et al. 1996; Deibel et al. 1996; Castellani et al. 1999).

We found that AD brain specimen had copper content similar to controls, reproducing previous (Platin et al. 1997) an recent results (Religa et al. 2006), even though other authors (Deidel et al. 1996) found decreased copper content in specific areas of the AD brain. The finding of a ceruloplasmin proteolysis in the brain supports the oxidative stress hypothesis. This evidence allows to suppose that a percentage of ceruloplasmin in the brain is not properly formed. Finally, it must be remarked that this study had a number of limitations which include: the fact that the AD samples used for the serum and brain analyses were from different individuals, resulting in a sampling bias; the relatively small size and amount of the brain samples; the lack of electron paramagnetic resonance (EPR) spectroscopy data to study the ceruloplasmin-copper binding affinity in AD.

## Figures and Tables

**Table 1:** Serum copper and ceruloplasmin in controls and AD patients.

	AD patients (n = 25)	Controls (n = 25)	test t
Serum Copper (μmol/L)	18 (6)	13.2 (2.2)	p < 0.001
Ceruloplasmin (mg/dL)	27.9 (6.2)	26.5 (4.3)	p = 0.369
Copper bound to ceruloplasmin (μmol/L)	13.1 (2.9)	12.5 (0.4)	p = 0.369
Non-ceruloplsmin-copper (NCC) (μmol/L)	4.9 (5.6)	0.7 (1.9)	p = 0.001

Data are presented as means (SD). Significant at the p = 0.05 level.

**Table 2:** Brain copper measurements in controls and AD patients.

	AD patients (n = 9)	Controls (n = 10)	test t
Total Protein (mg/mL)	0.79 (0.34)	1 (0.24)	p = 0.036
Copper/Protein (ng/mg wet weight)	5.126 (1.59)	6.77 (1.52)	p > 0.1

Data are presented as means (SD). Significant at the p = 0.05 level.
